# Longitudinal evaluation of serial ctDNA kinetics using joint modeling in patients with metastatic breast cancer

**DOI:** 10.1038/s41698-026-01498-8

**Published:** 2026-06-03

**Authors:** Christopher Pretz, Jesús Fuentes-Antrás, Sasha Main, Aaron Dou, Elizabeth Shah, Emily Van de Laar, Caroline M. Weipert, Bryan Lin, Amar Das, Eitan Amir, Michelle B. Nadler, Celeste Yu, Hal K. Berman, Lillian L. Siu, Philippe L. Bedard, Mitchell J. Elliott, David W. Cescon

**Affiliations:** 1 Guardant Health, Inc., Redwood City, CA USA; 2 NEXT Oncology, Hospital Universitario Quirónsalud Madrid, Madrid, Spain; 3 Department of Medical Biophysics, University of Toronto, Toronto, ON Canada; 4 Division of Medical Oncology & Hematology, Department of Medicine, Princess Margaret Cancer Centre, Toronto, ON Canada; 5 Cancer Genomics Program, Princess Margaret Cancer Centre, Toronto, ON Canada; 6 Department of Pathology and Laboratory Medicine, University Health Network, Toronto, Canada; 7 Division of Medical Oncology, Yale Cancer Center, New Haven, CT USA

**Keywords:** Biomarkers, Cancer, Computational biology and bioinformatics, Oncology

## Abstract

This study applies joint modeling (JM) of longitudinal and time-to-event data to circulating tumor DNA (ctDNA) dynamics measured by liquid biopsy in metastatic breast cancer (mBC), with a focus on dynamic prediction. Unlike traditional prognostic approaches that rely on static biomarkers, JM captures the continuous evolution of a methylation-based tumor fraction (TF) and links these changes to clinical outcomes. Serial liquid biopsies from 49 patients with hormone receptor–positive/HER2-negative mBC treated with endocrine therapy plus a CDK4/6 inhibitor were analyzed using the Guardant Reveal assay. JM combined a flexible longitudinal sub-model to characterize temporal TF trajectories with a Cox proportional hazards model for overall survival (OS) and time to treatment discontinuation (TTD). The most recent TF estimate was strongly associated with both OS and TTD (*p* < 0.0001), demonstrating an inverse relationship between TF and prognosis. These findings highlight the value of JM for generating patient-specific, dynamically updated predictions that may support real-time, personalized clinical decision-making in mBC management.

## Introduction

The utilization of joint modeling (JM) of longitudinal and time-to-event data has steadily increased since its inception, particularly in medical and epidemiological research^1^. The joint model is comprised of two sub-models: a longitudinal sub-model and a time-to-event sub-model, which are connected to capture the relationship between the two outcomes. JM has undergone significant improvements since it was first introduced^2–8^. Unlike traditional statistical approaches, which utilize a single baseline measure or differences between baseline and subsequent timepoints to predict cohort-level outcomes, JM is able to continuously monitor biomarker evolution and map how evolution modifies outcomes for a particular patient. Another distinct feature of JM is its capacity to accommodate endogenous time-varying covariates, contrasting with the conventional benchmarking time-dependent Cox-proportional hazard model, which assumes the time-varying biomarker is exogenous^9^. Furthermore, JM is able to generate patient-level dynamic predictions, with the potential to provide prognostic information that may inform patient outcomes and treatment response. As such, dynamic predictions are a focal point of this study. JM may also incorporate patient demographics, comorbidities, and other patient-specific factors to improve model accuracy, which may refine these predictions. While JM has been used previously in other breast cancer settings, including based on tumor size dynamics, little is known about the utility of JM and the incorporation of longitudinal measurements of analytes derived from liquid biopsy (LB)^10–12^.

LB facilitates the identification, quantification, and characterization of tumor-derived DNA fragments in the peripheral blood (circulating tumor DNA, ctDNA), where changes in ctDNA levels are associated with patient outcome. This study aimed to evaluate the validity of serial ctDNA measurements in JM in addition to increasing the awareness of this advanced statistical methodology. Herein, we demonstrate the utility of JM in a real-world cohort of patients with hormone receptor-positive/HER2-negative (HR+/HER2-) metastatic breast cancer (mBC) receiving endocrine therapy and a cyclin-dependent kinase 4/6-inhibitor (CDK4/6i) with serial ctDNA measurements to evaluate how tumor fraction (TF) evolution relates to time-to-event outcomes such as time to treatment discontinuation (TTD) and overall survival (OS).

## Results

### Study Cohort and data preparation

All participants were required to have stage IV disease as defined by the American Joint Committee on Cancer (AJCC) 8th edition prognostic staging system for breast cancer^13^. Sites of metastatic disease at enrolment were identified through manual review of diagnostic staging computed tomography (CT) scans. For cases of recurrent breast cancer, prior treatments and the interval between initial diagnosis and recurrence were recorded. Hormone receptor and HER2 status were determined on the most proximal pathology specimen prior to cohort enrolment (clinical diagnostic biopsy, if available, or the original surgical specimens for recurrent cases) and were reported per ASCO/CAP guidelines by institutional pathologists^14,15^. Patient characteristics, including age at diagnosis, sex, systemic therapies [(neo)adjuvant, palliative chemotherapy, targeted therapy, and/or endocrine therapy], endocrine therapy combination partners, specific CDK4/6 inhibitors (palbociclib, ribociclib, and/or abemaciclib), surgeries, and radiation treatments, were extracted through manual review of electronic health records. OS was defined as the time from the diagnosis of stage IV breast cancer to the date of death and TTD was defined as the duration from the initiation of ET + CDK4/6i therapy to its discontinuation due to progressive disease. Patients without an event were censored at their last follow-up, with treatment status recorded at that time. Treatment discontinuation and survival outcomes were last updated on September 23rd, 2024. Of the 59 patients in the cohort, 49 had a baseline and at least two additional longitudinal measures (279 total TF measures) and no missing covariate values, resulting in a complete case analysis (CCA) dataset. Baseline characteristics of the CCA dataset are described (Table 1), grouped by patient time to treatment discontinuation and survival status at the time of data cutoff. All analyses were performed using the CCA dataset.

**Table 1:** Cohort characteristics summarized by survival and treatment status for the complete case analysis dataset

Alive vs. Deceased			
Covariate		Alive (*n* = 30)	Deceased (*n* = 19)
Follow Up [Mean (range)], days		1062 (211–2008)	765 (102–1344)
Age [Mean (SD)]		61.8 (12.00)	62.4 (12.3)
CDK4/6 Inhibitor [Count (%)]			
	Palbociclib	21 (70.0%)	14 (73.7%)
	Abemaciclib or Ribociclib	9 (30.0%)	5 (26.3%)
Histology [Count (%)]			
	IDC	25 (83.3%)	15 (78.9%)
	ILC/MDLC	5 (16.7%)	4 (21.1%)
Line of Therapy [Count (%)]			
	First	24 (80.0%)	13 (68.4%)
	Two or More	6 (20.0%)	6 (31.6%)
Prior Adjuvant Treatment			
	Yes	13 (43.3%)	8 (42.1%)
	No	17 (56.7%)	11 (57.9%)
**Continued vs. Discontinued Treatment**			
**Covariate**		**Continued (***n* = **23)**	**Discontinued (***n* = **26)**
Follow Up [Mean (range)], days		930 (102-2008)	512 (82-1484)
Age [Mean (SD)]		59.9 (10.2)	63.87 (13.3)
CDK4/6 Inhibitor [Count (%)]			
	Palbociclib	16 (69.6%)	19 (73.1%)
	Abemaciclib or Ribociclib	7 (30.4%)	7 (26.9%)
Histology [Count (%)]			
	IDC	21 (91.3%)	19 (73.1%)
	ILC/MDLC	2 (8.7%)	7 (26.9%)
Line of Therapy [Count (%)]			
	First	20 (87.0%)	17 (65.4%)
	Two or More	3 (13.0%)	9 (34.6%)
Prior Adjuvant Treatment [Count (%)]			
	Yes	9 (39.1%)	12 (46.2%)
	No	14 (60.9%)	14 (53.8%)

*IDC* invasive ductal carcinoma, *ILC/MDLC* invasive lobular or mixed ductal and lobular carcinoma.

### Biomarker transformation and visualization

**Fig. 1:**
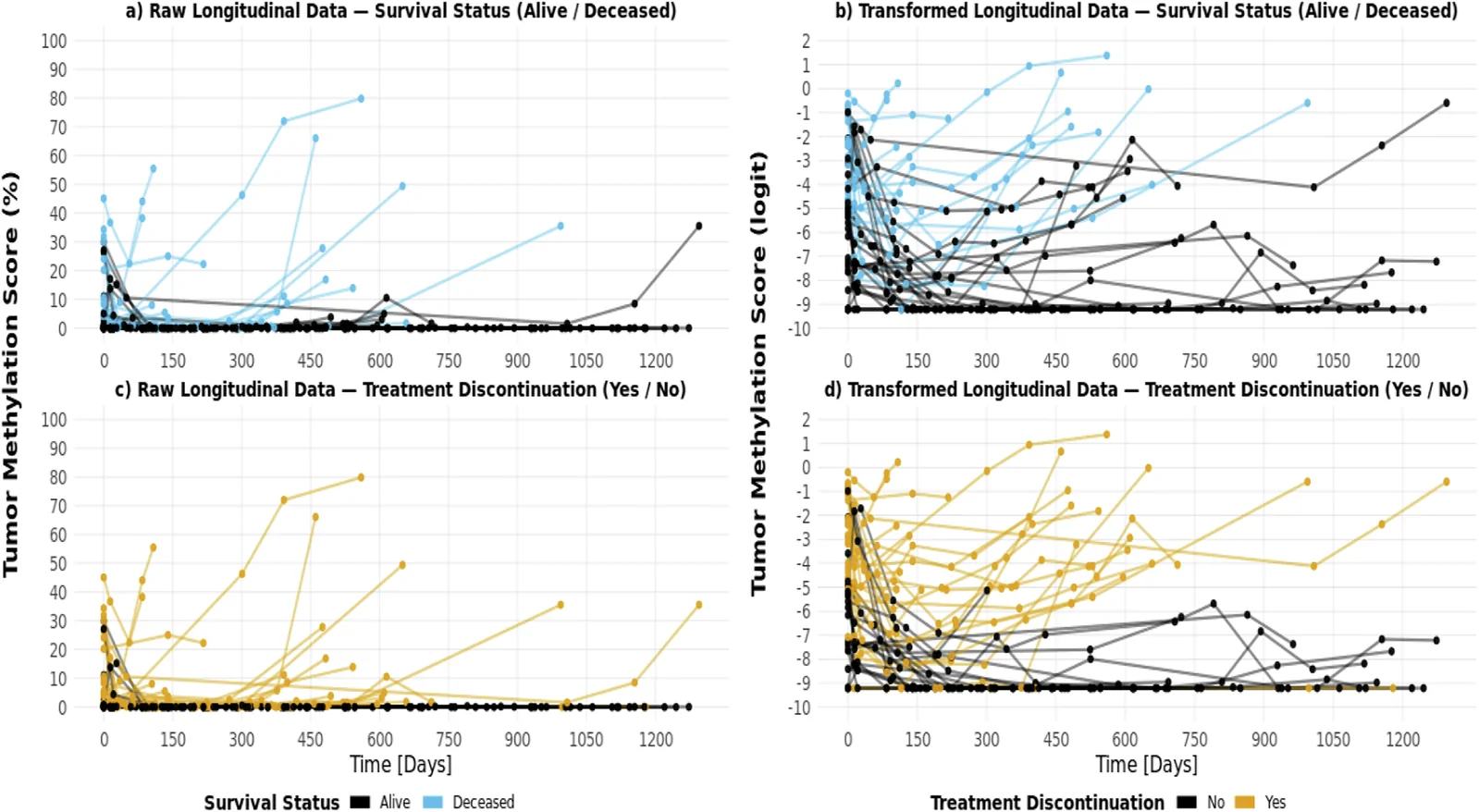
Spaghetti plots of raw and transformed longitudinal data for the cohort. Patients grouped by (**a**) Survival status and (**b**) Treatment Discontinuation.

A spaghetti plot of the raw and transformed temporal data, grouped by survival status and treatment discontinuation, are displayed below (Fig. 1). The spaghetti plot of the raw data shows that TFs are highly skewed where the vast majority of the temporal measures fall below a TF of 5% (Fig. 1a, c). In contrast, upon performing a logit transformation of the TFs (tTF), the skew becomes attenuated, which better conforms to model assumptions and the complex tTF evolution, both within and across patients, is revealed (Fig. 1b, d). Noting that this patient heterogeneity is what the longitudinal sub-model, a hierarchical cubic spline mixed effects model (HCSMEM), is adept at capturing. Details regarding the HCSMEM, and how it was selected are discussed in the Methods section. Plots of the transformed data also show that the temporal trajectories differed between participants who experienced and did not experience an OS event in the follow-up period (Fig. 1b) and those who experienced treatment discontinuation versus those who did not (Fig. 1d). These between-group discrepancies play an important role within the JM architecture in informing patient-level dynamic predictions.

A Cox proportional hazards (CPH) model i.e. the time-to-event sub-model, was used to assess OS and TTD. In executing the JM, a Bayesian approach was adopted to determine the relationship between sub-models, as this approach offers more parameterization options compared to frequentist approaches^1^. Among the association structures explored (see the methods section for a detailed account of these structures), both the current tTF value and the time-averaged cumulative effect (TACE) demonstrated highly significant associations with the time-to-event outcomes (*p* < 0.00001), which in a Bayesian JM framework, corresponds to posterior probabilities of association approaching 1.00. Although the JMs incorporating the TACE demonstrated a more optimal fit as indicated by lower Watanabe–Akaike information criterion (WAIC) values, the deviance information criteria (DIC) suggest that the JMs with the most current biomarker value exhibit better fit (Supplementary Table 1). Ultimately, the models based on the current tTF estimate were selected for dynamic prediction generation due to greater clinical interpretability. The results of the JMs based on the most recent tTF estimate for OS (Table 2) and TTD (Table 3) are summarized. Model results for the JMs based on the TACE can be found in the Supplementary Tables 2 and 3.

**Table 2:** Joint model results for the time-to-event sub-model (Overall Survival—Current tTF Value)

Time-to-event jm results for overall survival (Current tTF Value)						
Covariate	Parameter Estimate (HR)	SD of Estimate	95% CI (LB, UB)	HR 95% CI (LB, UB)	*p* value	R-hat
Baseline Age	0.008 (1.01)	0.042	(−0.075, 0.091)	(0.93, 1.10)	0.84	1.020
CDK4/6 Inhibitor: Palbociclib vs Abemaciclib or Ribociclib	−0.900 (0.41)	1.03	(−3.04, 1.02)	(0.048, 2.77)	0.38	1.080
Histology: IDC vs ILC/MDLC	−0.590 (0.55)	0.85	(−2.40, 0.95)	(0.091, 2.59)	0.50	1.005
Line of Therapy: 1st vs 2+	−0.480 (0.62)	0.72	(−1.97, 0.87)	(0.139, 2.39)	0.52	1.020
Prior Adjuvant Treatment (No vs Yes)	−0.60 (0.55)	0.70	(−2.02, 0.73)	(0.133, 2.08)	0.38	1.001
Current Value (tTF)	0.52 (1.69)	0.15	(0.263, 0.84)	(1.30, 2.32)	<0.0001	1.020

**Fig. 2:**
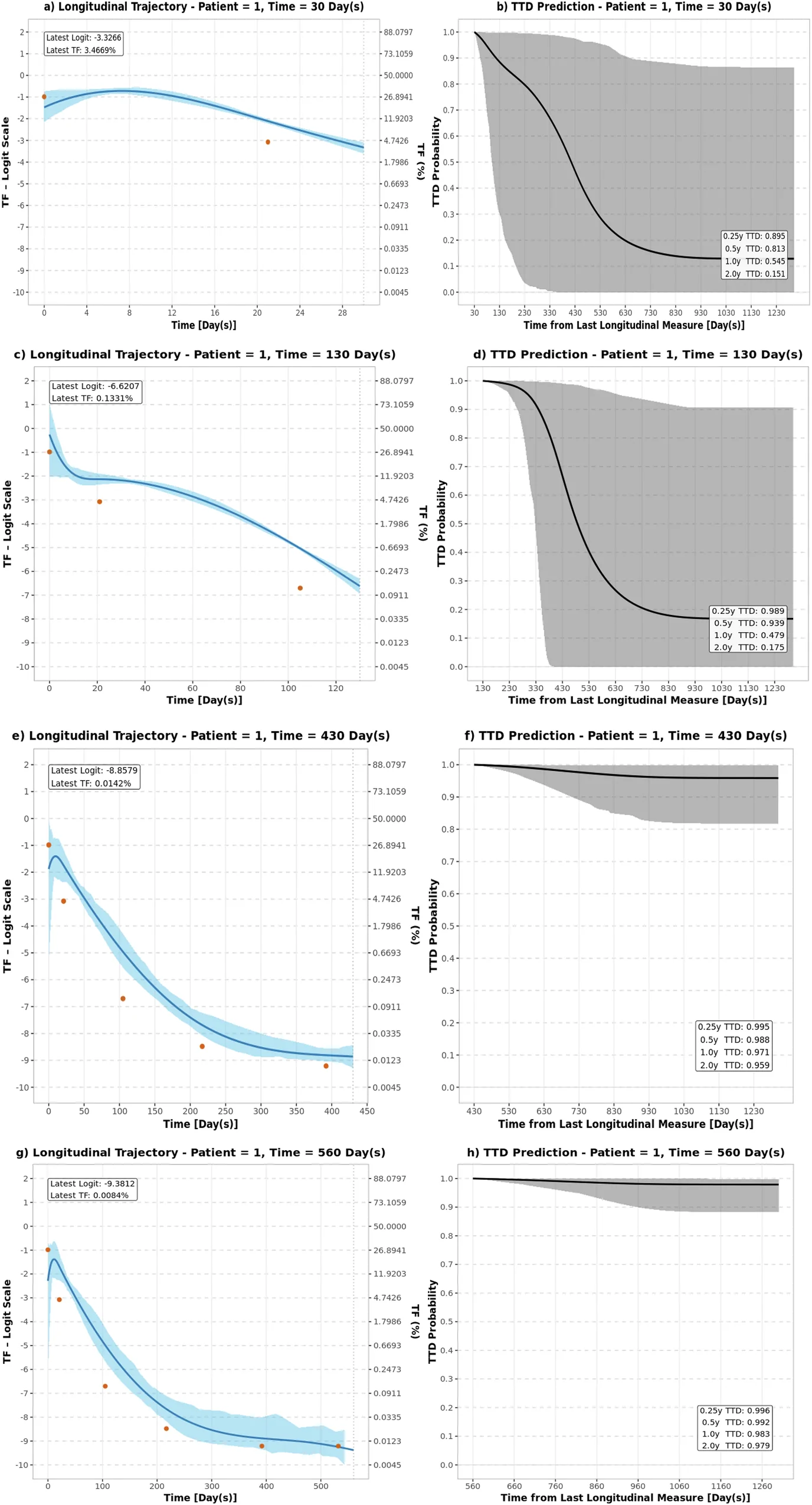

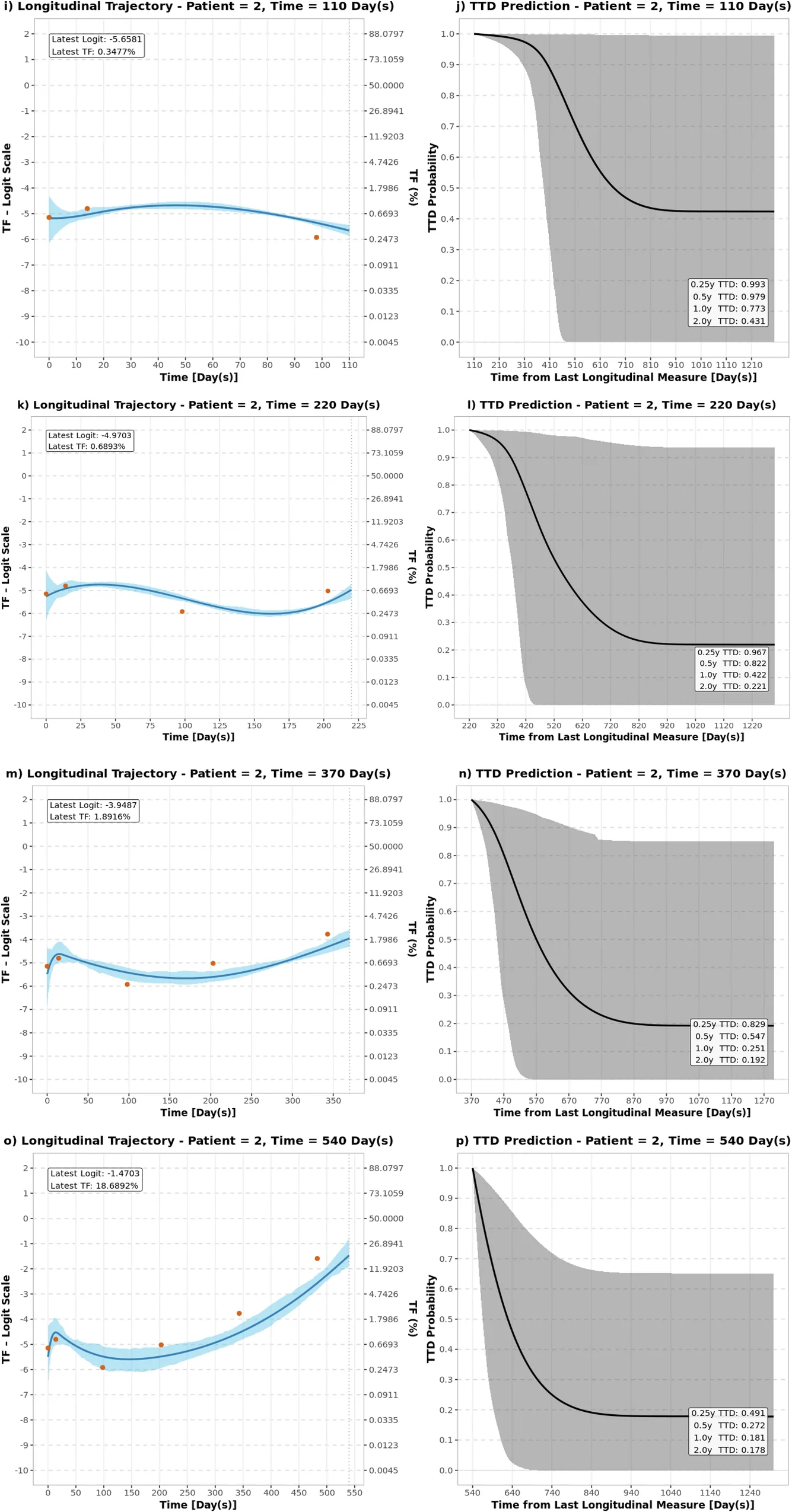
Dynamic predictions of time to treatment discontinuation evolve based on serial monitoring of tumor fraction. Predicting time to treatment discontinuation (TTD) survival probabilities based on the current estimated transformed tumor fraction (tTF) for patient 1 (**a**–**h**) and patient 2 (**i**–**p**). Figures **a**, **c**, **e**, **g**, **i**, **k**, **m**, and **o** show the trajectory of the patient’s estimated tTF level where these estimates are driven by the existing observed tTF pattern (given by the orange dots). The most recent tTF estimate is reported in the upper left-hand corner of the plot and is updated each time the tTF is re-estimated. Figures **b**, **d**, **f**, **h**, **j**, **l**, **n** and **p** show the patient’s TTD prediction where the specific projected 0.25-, 0.5-, 1.0- and 2.0-year event probabilities are reported in the bottom right-hand corner of the TTD prediction plots (**b**, **d**, **f**, **h**, **j**, **l**, **n**, **p**). TTD predictions are updated each time the tTF is re-estimated and are informed by the most recent observed tTF measurement.

*SD* Standard Deviation, *CI* Credible Interval, *LB* Lower Bound of the CI, UB Upper Bound of the CI, IDC invasive ductal carcinoma, ILC/MDLC invasive lobular or mixed ductal and lobular carcinoma. Note that since cubic spline model parameter estimates are uninterpretable, model results are displayed graphically (see longitudinal sub-model results (Fig. 2)).

**Table 3:** Joint model results for the time-to-event sub-model (time to treatment discontinuation—current tTF value)

Time-to-event JM Results for Time to Treatment Discontinuation (Current tTF Value)						
Covariate	Parameter estimate (HR)	SD of estimate	95% CI (LB, UB)	HR 95% CI (LB, UB)	*p*-value	R-hat
Baseline Age	0.019 (1.02)	0.033	(−0.046, 0.084)	(0.96, 1.09)	0.56	1.001
CDK4/6 Inhibitor: Palbociclib vs Abemaciclib or Ribociclib	−0.521 (0.59)	0.79	(−2.12, 0.98)	(0.12, 2.66)	0.52	1.003
Histology: IDC vs ILC/MDLC	0.029 (1.03)	0.614	(−1.22, 1.19)	(0.30, 3.29)	0.94	1.001
Line of Therapy: 1st vs 2+	0.307 (1.36)	0.55	(−0.79, 1.36)	(0.45, 3.90)	0.57	1.001
Prior Adjuvant Treatment (No vs Yes)	−0.261 (0.77)	0.56	(−1.37, 0.49)	(0.25, 1.63)	0.64	1.002
Current Value (tTF)	0.723 (2.06)	0.13	(0.49, 1.01)	(1.64, 2.74)	<0.0001	1.020

**Fig. 3:**
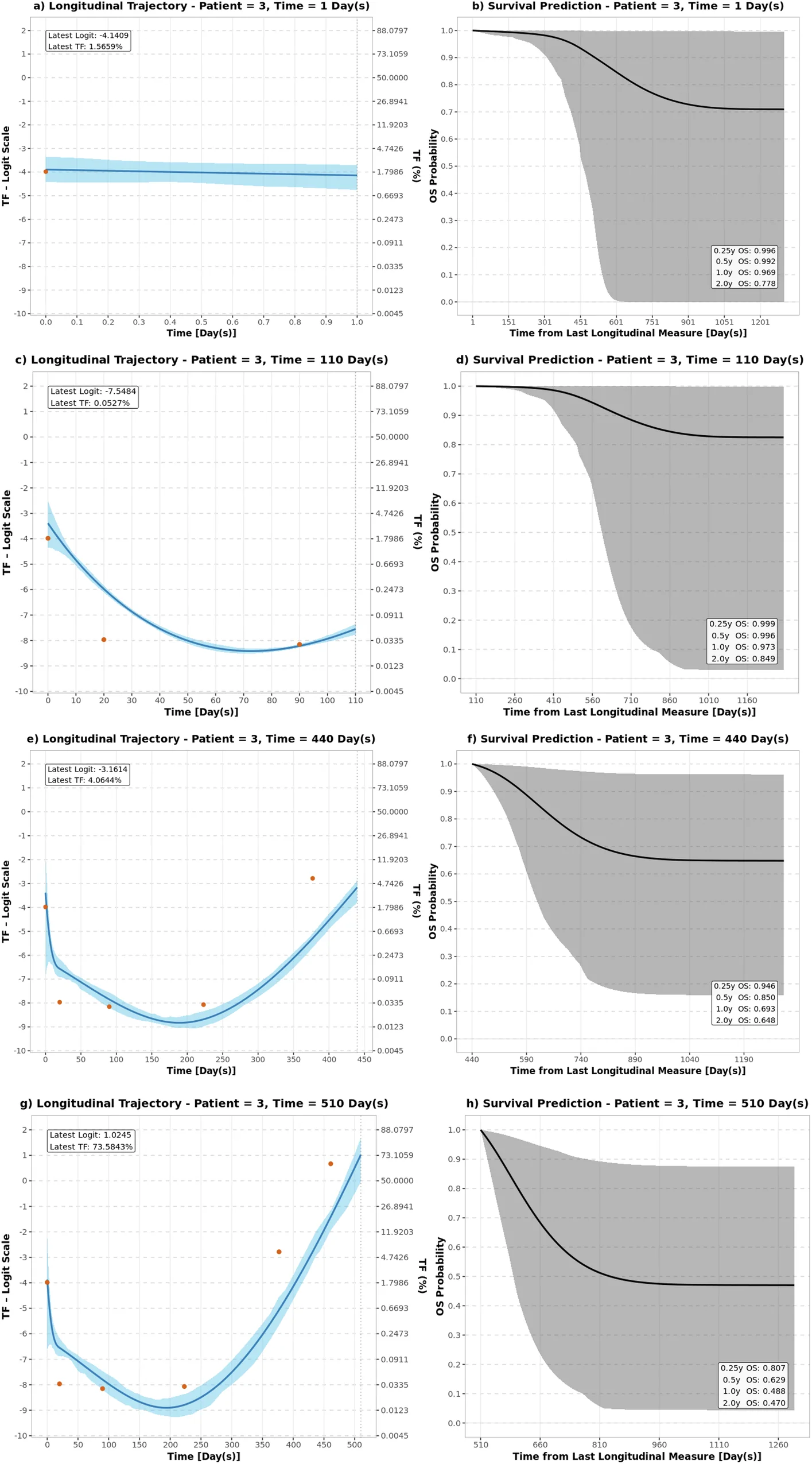

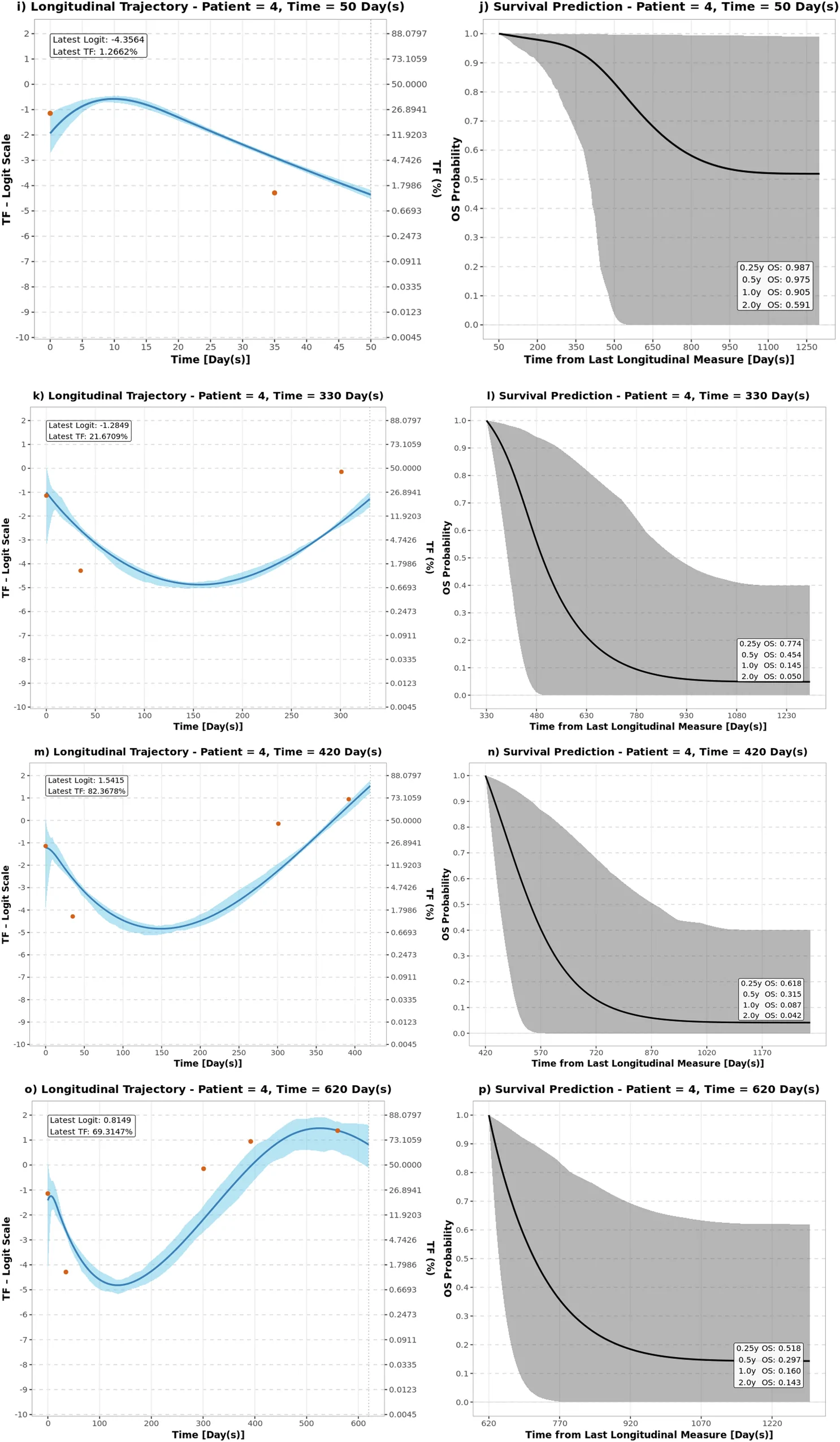
Dynamic predictions of overall survival evolve based on serial monitoring of tumor fraction. Predicting overall survival (OS) probabilities based on the current estimated transformed tumor fraction (tTF) for patient 3**a**–**h** and patient 4 (**i**-**p**). Figures **a**, **c**, **e**, **g**, **i**, **k**, **m**, and **o** show the trajectory of the patient’s estimated tTF level, where these estimates are driven by the existing observed tTF pattern (given by the orange dots). The most recent tTF estimate is reported in the upper left-hand corner of the plot and is updated each time the tTF is re-estimated. Figures **b**, **d**, **f**, **h**, **j**, **l**, **n**, and **p** show the patient’s OS prediction where the specific projected 0.25-, 0.5-, 1.0-, and 2.0-year event probabilities are reported in the bottom right-hand corner of the OS prediction plots (**b**, **d**, **f**, **h**, **j**, **l**, **n**, **p**). OS predictions are updated each time the tTF is re-estimated and are informed by the most recent observed tTF measurement.

*SD* Standard Deviation, *CI* Credible Interval, *LB* Lower Bound of the CI, *UB* Upper Bound of the CI, *IDC* invasive ductal carcinoma, *ILC/MDLC* invasive lobular or mixed ductal and lobular carcinoma. Note that since cubic spline model parameter estimates are uninterpretable, model results are displayed graphically (see longitudinal sub-model results (Fig. 3)).

### Hazard ratios and clinical interpretability

The output in Tables 2 and 3 can be interpreted similarly to a traditional CPH model with hazard ratios (HR) estimated by exponentiating parameter estimates. For example, since the focus was on the biomarker’s most recent value, after controlling for confounders, a one-unit logit increase in the estimated tTF translates into a 1.69 [exp(0.52)] fold increase in the patient’s risk of experiencing death at a given time point. Importantly, this HR represents a global association parameter, meaning that it reflects a common proportional effect across all patients and timepoints. This result suggests that OS outcomes tend to improve as the most recent tTF estimate decreases, and conversely, higher tTF estimates are associated with less favorable outcomes. Furthermore, based on the results in Table 2, a similar relationship is expected for TTD. Since parameter estimates of cubic spline models are not directly interpretable^16–18^, spline model parameter estimates for the longitudinal sub-model are not reported. Instead, we display the modeled longitudinal trajectories graphically in Figs. 2 and 3.

### Dynamic patient-level predictions

While the HR is useful for capturing an overall trend, given that the primary objective is to demonstrate a patient-centric application of the JM, we now focus on patient-level dynamic predictions, where graphical depictions for two different select patients for TTD (Fig. 2) and OS (Fig. 3) are provided. Dynamic prediction processes are illustrated by pairing patient-specific tTF trajectories with survival probabilities that are explicitly conditional on the patient’s longitudinal biomarker estimate at the time of prediction (Figs. 2 and 3). Each pair of panels provides a visualization of the evolving patient longitudinal profile alongside the associated probability of not experiencing the outcome, with 95% credible intervals indicating model uncertainty. In the longitudinal trajectory panels, the tTF estimates (smooth blue curve) are obtained by fitting the longitudinal sub-model to observed tTF measurements (orange dots). As additional blood draws become available, the estimated tTF values are updated accordingly. Notably, as these modeled trajectories interpolate between and extrapolate beyond observed measurements, continuous estimates of the tTF are derived, enabling dynamic prediction of time-to-event outcomes at any given time point. To enhance result interpretation, back-transformed tTF values are also provided. Dynamic predictions are typically generated for a "new" patient who is assumed to be event free at the time of assessment, although previously observed longitudinal information from an existing patient can also be used.

### Interpretation of dynamic predictions and prediction horizons

**Fig. 4:**
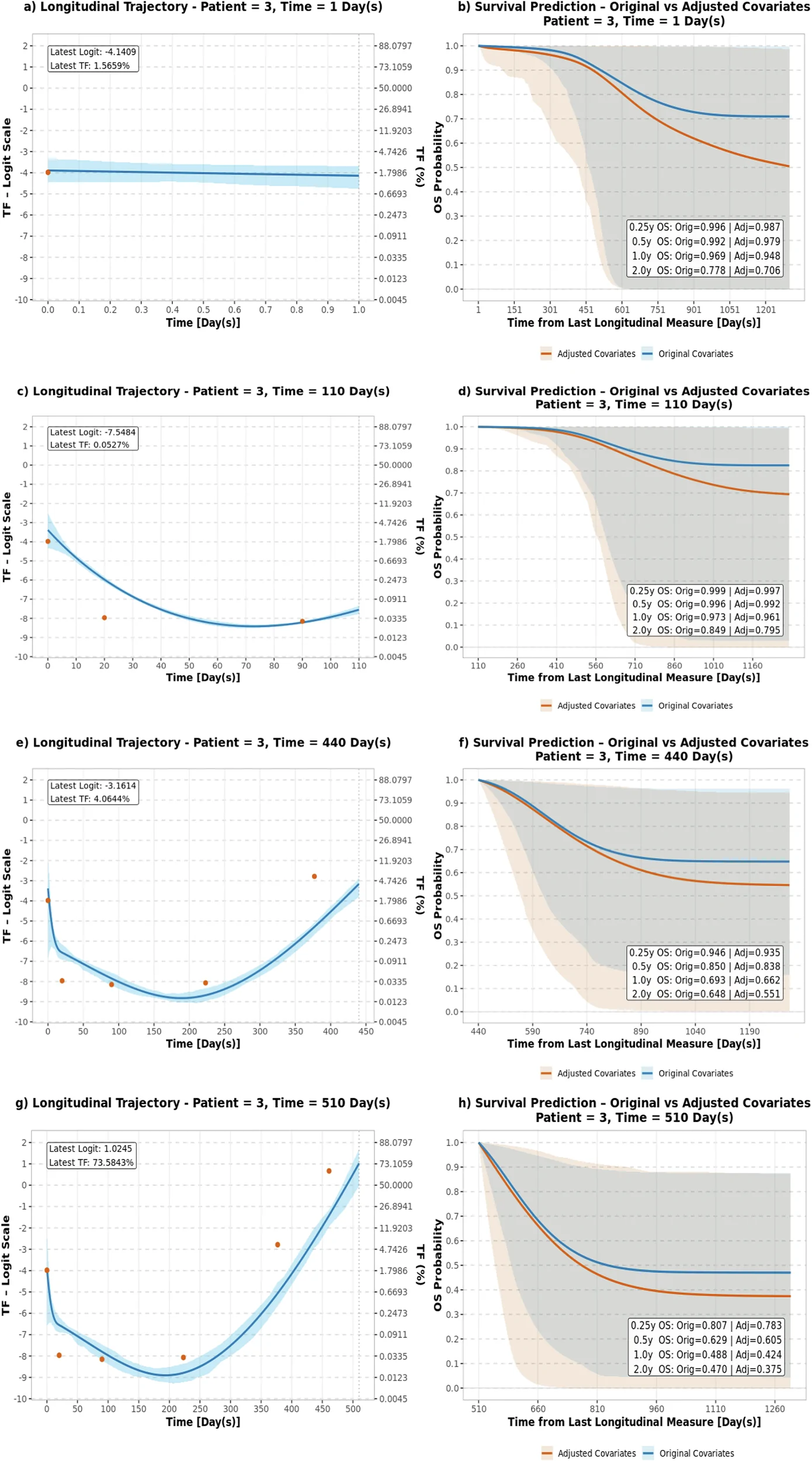
Dynamic predictions of overall survival for a patient with identical TFs but different baseline clinical characteristics. Dynamic predictions of patient OS for patient 3 with original covariate values (blue survival curve; 59-year-old individual receiving Palbociclib, with a history of adjuvant therapy, currently receiving first-line ET + CDK4/6i treatment, and diagnosed with ILC/MDLC) and adjusted covariate values (orange survival curve; 79-year-old individual receiving ribociclib, with no history of adjuvant therapy, currently on second-line treatment, and diagnosed with IDC) baseline clinical characteristics. The projected 0.25-, 0.5-, 1.0-, and 2.0-year event probabilities are reported in the bottom right-hand corner of the OS prediction plots (**b**, **d**, **f**). Orange dots = tTF values, and the latest TF estimates (logit scale and percentage) are reported in the upper left-hand corner of the longitudinal trajectory plots (**a**, **c**, **e**).

In our analysis, survival probabilities are dynamically updated based on the most recent tTF estimate, and the survival curve is effectively reset each time the latest biomarker value is re-estimated. The value of the most recent tTF estimate (reported on the logit scale), and the back-transformed tTF (given as a percent), are presented in the upper left-hand corner of the longitudinal trajectory plots (Figs. 2–4). Although prediction horizons are necessarily user-defined, we selected clinically interpretable horizons of 0.25, 0.5, 1, and 2 years to facilitate comparison across time points. The probabilities associated with these horizon times are displayed in the lower right-hand corner of survival prediction plots (Figs. 2–4).

To illustrate the dynamic prediction process, consider patient 1. For this patient the estimated tTF at 30 days is -3.3 logits (TF = 3.47%) (Fig. 2a), which corresponds to the TTD probabilities (i.e. not experiencing treatment discontinuation) of 0.9, 0.81, 0.55, and 0.15 at 0.25 y, 0.5 y, 1.0 y, and 2.0 y respectively (Fig. 2b). As additional longitudinal data is captured, the patient’s estimated tTF at 130 days decreases to -6.6 logits (TF = 0.133%, Fig. 2c), where the corresponding TTD probabilities improve (Fig. 2d). The TTD probability at one year actually decreased from 0.55 to 0.48 due to the non-linear nature of the survival curve, but in general, the inverse relationship between the most recent tTF estimate and TTD probably holds. This trend is demonstrated as the estimated tTF values declined further at later time points (Fig. 2e, g), with continual improvements in projected outcomes (Fig. 2f, h) being observed across different time horizons. This pattern reflects the expected negative relationship between the most recent biomarker value and the associated probabilities extracted from the survival curves, as similar trends are observed for patients 2 (Fig. 2)3, and 4 (Fig. 3), further supporting the consistency and robustness of this association between decreasing tTF estimates and improved projected TTD and OS outcomes.

### Impact of baseline patient characteristics

Dynamic predictions derived from JMs have demonstrated concordance with expected clinical patterns. Subsequent analyses focused on evaluating the impact of individual patient characteristics on predicted outcomes. A key advantage of the JM framework is its ability to incorporate covariates into both sub-models. This feature allows for the adjustment of potential confounders and enhances the accuracy of individualized predictions. To demonstrate this capability, survival curves were contrasted for a single patient while holding the longitudinal biomarker profile fixed and varying only the covariate values used in the survival sub-model. Specifically, outcomes for patient 3, a 59-year-old individual receiving palbociclib, with a history of adjuvant therapy, currently on first-line ET + CDK4/6 treatment and diagnosed with invasive lobular or mixed ductal–lobular carcinoma (ILC/MDLC), are shown using the original covariate values (Fig. 4, Original Covariates). These results are compared with predictions generated under an alternative covariate profile representing a 79-year-old patient receiving ribociclib, with no history of adjuvant therapy, currently on second-line treatment, and diagnosed with invasive ductal carcinoma (IDC) (Fig. 4, Adjusted Covariates). These findings underscore that while the most recent tTF estimate is a critical driver of survival predictions, it does not operate independently of other patient-specific factors. The observed decline in prognosis using the adjusted covariates is consistent with clinical expectations, given the older age and more advanced line of therapy; however, additional clinical interpretation may be warranted to contextualize the influence of other covariates.

### Model validation

The predictive performance of each JM is evaluated based on a matrix of Brier Scores (BrSc), where the advantages of using this particular model accuracy metric are presented in the methods section. Each BrSc matrix is generated by iterating over various start and time horizon combinations, selected to capture the predictive landscape of each model. The BrSc are then fit to a three-dimensional surface to form a continuous prediction space. Heatmaps are generated from these surfaces to visually summarize predictive accuracy across the range of clinically relevant forecasting intervals. As lower BrSc values indicate better prediction, regions of the heatmap approaching zero reflect greater accuracy. Table 4 provides reference thresholds and interpretive guidance for BrSc values^19–21^.

**Table 4:** Summary of Brier score interpretations

Brier Score Value (at time *t*)	Interpretation in Survival Context	Discussion
0.00	Perfect prediction	Predicted survival exactly matches observed survival (rare in practice).
≤ 0.05	Excellent predictive accuracy	Strong calibration and discrimination at time *t*.
0.05 – 0.15	Good predictive performance	Useful clinical utility if consistently low across relevant follow-up times.
0.15 – 0.25	Moderate predictive performance	Model provides some predictive value but has weaker calibration/discrimination.
≈ 0.25	Random guess equivalent	At ~ 50% survival probability, performance is no better than chance.
> 0.25	Poor predictive accuracy	Worse than a naïve baseline (e.g., Kaplan-Meier prediction).

**Fig. 5:**
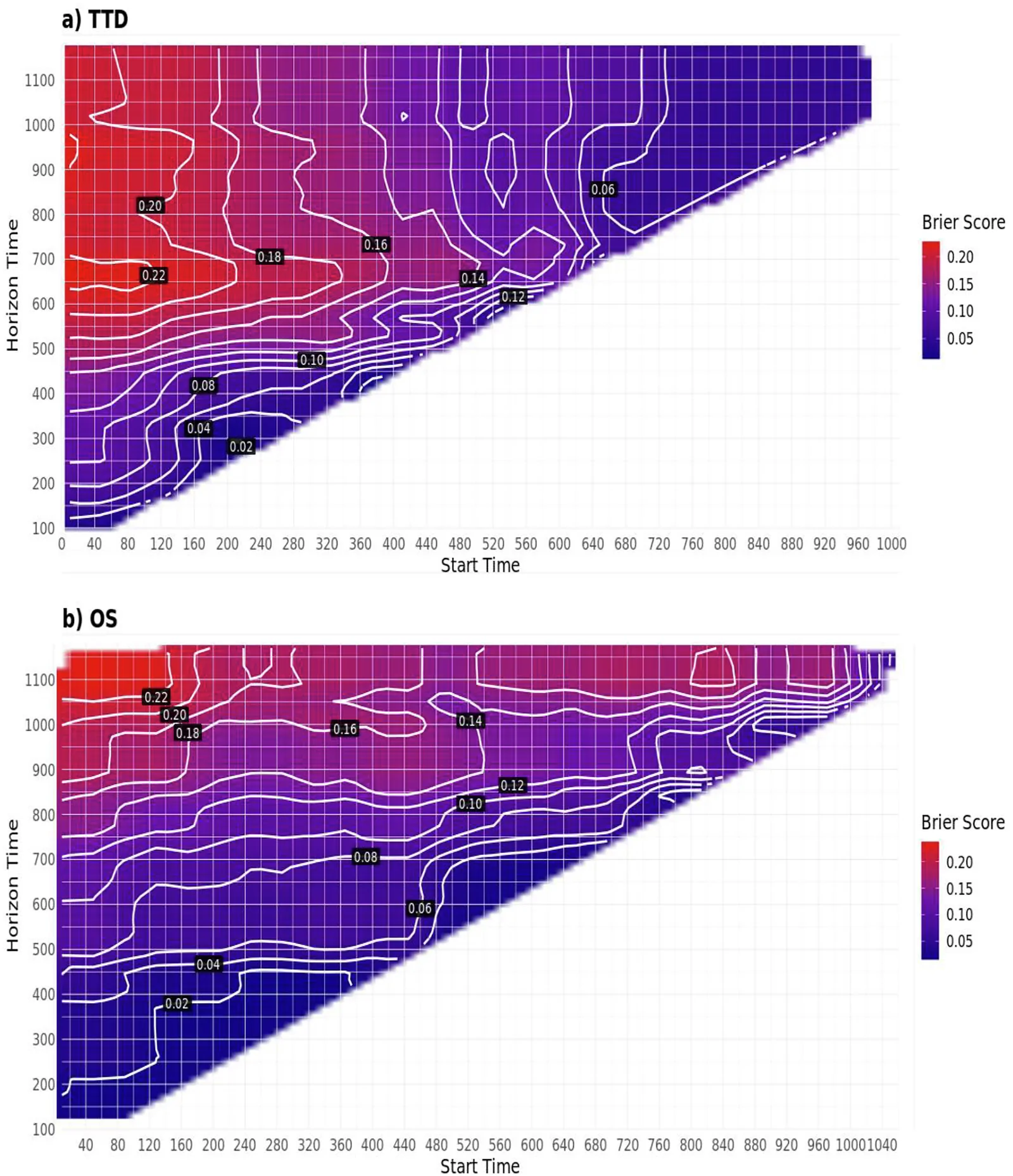
Time-Dependent Brier Score Heatmaps Illustrating Predictive Performance of Joint Models Across Start/Horizon Time Combinations. **(a)** TTD and **(b)** OS Brier Score Heatmaps for each respective joint model. The x-axis represents the start time and the y-axis represents the horizon time. Heatmap colors correspond to Brier score magnitude, where lower (blue) values indicate better predictive performance. White contour lines represent transitions in Brier score values.

The BrSc heatmaps for each JM (Fig. 5a, b) demonstrate predictive accuracy ranging from excellent to moderate (Table 4). These heatmaps provide an intuitive, continuous visualization of model behavior across the full complement of start and horizon times. To enhance interpretability, contour lines are overlaid to demarcate transitions between levels of accuracy. For example, based on the tTF estimate at 80 days, and, by predicting across all patients, the model predicts OS at 210 days with a BrSc of 0.02 (as shown by the contour lines in Fig. 5b). However, as the horizon time increases to 390 days, the BrSc increases to 0.04. This increasing trend continues, with predictions falling within the “good predictive performance” range up to horizon time of approximately 810 days, beyond which BrSc values enter the lower bound of the “moderate” range—reflecting the expected increase in uncertainty for more distant forecast horizons. Although the predictive landscape for OS appears more favorable than that for TTD, both heatmaps collectively show that each model maintains acceptable levels of accuracy across most clinically relevant start–horizon combinations. In particular, each model consistently achieves accuracy within the excellent-to-good range for the majority of the prediction windows examined.

A caveat to interpreting the heatmaps is that the same dataset was used for both model estimation and performance assessment—which can lead to optimistically biased BrScs. To quantify and correct for this bias, we applied the bootstrap-based optimism adjustment proposed by Andrinopoulou et al. (2021)^22^. Because this procedure is computationally intensive—requiring repeated refitting of each joint model—we evaluated the optimism using a subset of start–horizon time combinations intended to represent the broader prediction landscape shown in the heatmaps.

**Fig. 6:**
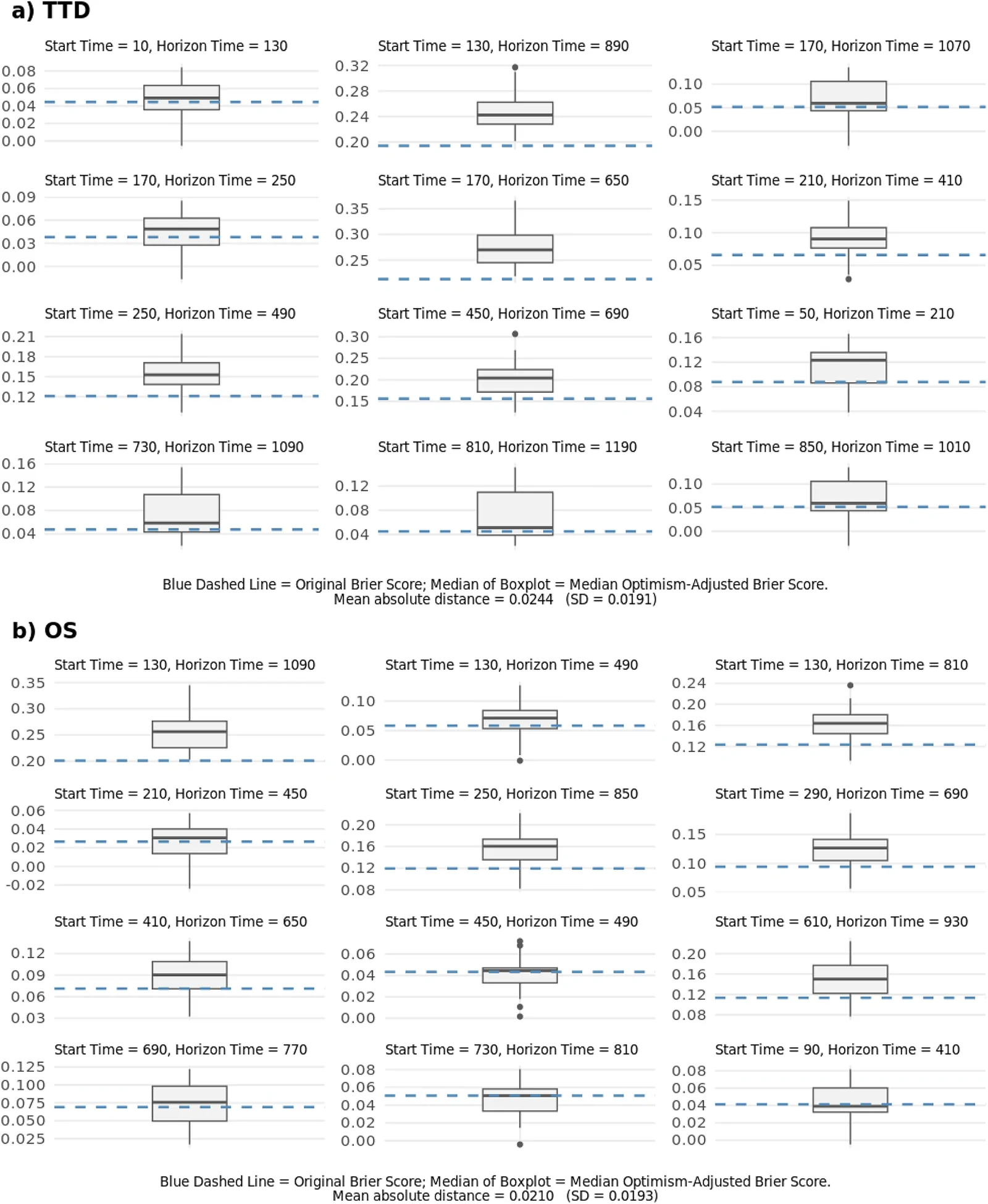
Bootstrap 0ptimism-adjusted Brier score box-plots for selected start/horizon time combinations. **(a)** TTD and **(b)** OS optimisim-adjusted Brier score box-plots comparing bootstrap-corrected estimates with their naive Brier score values across representative start/horizon time combinations. Blue dashed lines denote the naive Brier score estimates while box-plots summarize the distribution of the optimism-adjusted Brier score values across bootstrap resamples. Mean absolute distances represent the average difference between the naive and median optimism-adjusted Brier score estimates.

For each selected combination within each JM, the distribution of optimism-adjusted BrScs is displayed as boxplots (Fig. 6a, b), with the blue dashed line representing the naïve i.e., original optimistic estimate. Across all evaluated combinations, the bootstrapped realizations show modest upward adjustment, indicating that some optimism is present but generally limited in magnitude. Although the gap between the naïve estimate and the bootstrap median tends to widen for longer horizon times, the medians largely remain within the same qualitative performance categories defined (Table 4), representing excellent to good performance. On average, the absolute difference between the naïve estimate and the corresponding bootstrap median is 0.024 (SD = 0.019) for TTD and 0.021 (SD = 0.019) for OS, suggesting that the joint models retain robust predictive performance even after accounting for potential overfitting. The narrow interquartile ranges observed in the boxplots further support the stability of these bias-corrected estimates.

## Discussion

This study applied JM to combine longitudinal TF measurements derived from ctDNA-based assays in patients with HR+/HER2− mBC with patient outcomes. The analysis demonstrated that JM successfully leveraged repeated measures of an epigenomic biomarker, captured via liquid biopsy, to generate individualized survival predictions that account for both dynamic molecular changes and fixed baseline patient characteristics.

By transforming TF to the logit scale, we addressed the skewed distribution commonly seen in percentage-based molecular readouts and improved the suitability of the data for employing the HCSMEM, designed to capture both between and within-patient variability as seen in the transformed data. Differences in longitudinal trajectories were observed between those who experienced OS events and those who did not, as well as between those who discontinued treatment and those who remained on therapy. These differences were captured effectively using a Bayesian JM framework that jointly estimated the biomarker trajectory and the time-to-event projections, allowing for statistical adjustment for relevant clinical covariates. The association between higher tTF values and increased event risk was consistent across both OS and TTD, reinforcing the prognostic relevance of serial ctDNA-derived TF in mBC and supporting its integration into risk assessment strategies.

A feature of JM is its ability to generate dynamic, patient-level predictions that update as new data become available. Survival probabilities evolved over time with the incorporation of additional tTF measurements, highlighting the temporal relationship between declining biomarker levels and improving prognosis, and conversely, with rising tTF value and an increasing likelihood of experiencing an event. Importantly, the dynamic predictions were influenced not only by the most recent tTF value but also by the broader clinical context of each patient. Importantly, as seen through the JM, this highly significant association reflects a relationship that holds across all patients and throughout the entire observation window, meaning the highly significant p-values (Tables 2–3) pertains to a longitudinal effect that not only pertains to each patient, but is also relevant continuously over time. While the evolving trajectory of the biomarker played a central role in shaping risk estimates, static clinical covariates meaningfully modulated these predictions. These findings highlight the necessity of accounting for patient-level heterogeneity when modeling time-to-event outcomes using molecular data. Unlike in a landmark CHP model, where the HR reflects an effect based on a single fixed timepoint, the HR in a JM reflects how each patient’s continuously evolving biomarker trajectory informs their risk over time. Thus, the JM updates each patient’s risk profile whenever a new biomarker estimate becomes available, while simultaneously accounting for measurement error, informative dropout, and covariate adjustment.

Model performance was evaluated using the time-dependent BrScs over a grid of start/horizon times. For many clinically relevant regions of the grid (start times up to ~600 days and horizon times up to ~700–800 days), BrSc values for both TTD and OS remained low, with most values between 0.02 and 0.10, where for long-term predictions, the BrSc values increased to 0.15–0.20, an expected result since projections into the distant future are often more difficult to conduct. BrScs generated herein compare favorably with those reported in other dynamic prediction settings. For example, Devaux et al.^23^ reported mean BS of 0.076–0.089 for 3-year mortality prediction in primary biliary cholangitis using high-dimensional landmark models, and Rizopoulos and Taylor^24^ reported integrated BrScs around 0.072–0.078 for joint PSA–recurrence models in a large prostatectomy cohort. Despite our relatively limited sample size, the BrScs obtained are within similar ranges, suggesting that both OS and TTD JMs provide acceptable levels of dynamic prediction accuracy. Finally, we demonstrate that the bias which arises from using the same data to train and test the model has a negligible impact on results.

Traditional approaches, such as time-dependent Cox models, assume that the longitudinal covariate is exogenous. However, in many clinical contexts, particularly when a biomarker is influenced by disease progression or treatment response, this assumption does not hold. JM addresses this limitation by properly modeling the biomarker process and the event time, thereby accounting for the endogenous relationship between the two. This is especially relevant for ctDNA, which enables serial monitoring of tumor burden and treatment response throughout a disease course. Current standard-of-care methods for monitoring disease in mBC rely heavily on cross-sectional imaging and in some cases, serum markers (i.e. CEA, CA15-3). Both approaches have limitations, including low sensitivity, infrequent measurement intervals, and logistical challenges. In contrast, leveraging LBs offers the possibility of higher-frequency, tumor-specific, high-sensitivity and minimally invasive monitoring, with growing evidence of clinical utility. Studies such as PADA-1, SERENA-6, and RADIOHEAD have demonstrated that on-treatment ctDNA dynamics are associated with clinical outcomes, can be detected prior to clinical endpoints, and may inform treatment adaptation leading to improved outcomes^25–27^. These findings, in combination with the modeling framework described here, support the broader development of ctDNA-based therapy monitoring approaches.

The JM framework offers flexibility in model structure and application. Although this study used a CPH sub-model for the event process and a hierarchical mixed-effects model for the biomarker trajectory, JM can be extended to incorporate multiple biomarkers, non-Gaussian longitudinal processes, and alternative event models such as accelerated failure time models^28,29^. Additionally, different association structures, such as time-dependent slopes or TACE, may be more appropriate in other clinical contexts. Recent advances in JM modeling approaches, such as super learner JMs, provide further opportunities to enhance predictive accuracy, particularly when multiple longitudinal signals are available^30^. Finally, future investigation should also focus on external validation in larger, more diverse patient populations and across different tumor subtypes and therapeutic contexts. Such efforts will be critical to demonstrating that JM-based dynamic predictions can inform treatment decisions in real time and to establishing their broader clinical utility beyond the scope of the current study.

While these data demonstrate the potential clinical utility of JM for longitudinal assessment, they should not be interpreted as suggesting that clinicians act on tumor fraction changes in isolation. Rather, this framework provides a structured approach to contextualizing serial ctDNA dynamics alongside standard radiologic, clinical, and symptomatic assessments. By generating individualized, time-updated risk estimates, joint modeling may enable earlier identification of patients at increased risk of progression than would be possible with single time-point assessments, help provide reassurance when tumor fraction is declining despite equivocal or indeterminate imaging, and improve prognostic clarity for patients experiencing prolonged response on ET plus CDK4/6 inhibition. In practice, such dynamic predictions may inform the intensity and timing of clinical surveillance, including radiologic de-escalation in patients with sustained molecular response or closer monitoring and consideration of earlier treatment switch or treatment intensification strategies in those with unfavorable ctDNA trajectories. Prospective validation studies will be necessary before joint model-derived predictions can be considered for use in clinical decision-making. In future implementations, JM-derived predictions could be incorporated into assay reports as intuitive, time-updated risk summaries or trajectory visualizations, facilitating clinical interpretation and integration into routine oncologic decision-making without requiring familiarity with the underlying statistical framework.

This study has several limitations. Firstly, the sample size was limited, which contributed to the variability in the predicted survival probabilities and wider credible intervals around some estimates. While convergence diagnostics were acceptable, more robust parameter estimates would be expected from larger datasets. The cohort was derived from a single academic cancer center and included only patients with HR+/HER2- mBC receiving ET and a CDK4/6i, potentially limiting generalizability to other subtypes and/or therapeutic settings. Patients were also required to have at least three ctDNA measurements to be included, which excluded those with rapid progression or early loss to follow-up, which may have introduced selection bias favoring patients with more indolent disease or better performance status. Although clinical covariates were incorporated and multiple association structures were examined, unmeasured confounders may still influence both ctDNA dynamics and time-to-event outcomes. As a result, subsequent work should focus on improving individualized risk stratification through the potential refinement of model architecture by integrating additional genomic features such as specific mutations and methylation or fragmentomic signatures, which may further refine dynamic risk evaluation. Similarly, enhancing the temporal resolution of ctDNA sampling and exploring multimodal data inputs, such as imaging or clinical response metrics, may further strengthen the predictive performance of JM in real-world settings.

In conclusion, this study provides a framework for integrating longitudinal TF dynamics with time-to-event outcomes using JMs, offering insight into real-time, personalized risk prediction in mBC. By coupling biomarker trajectories with baseline patient characteristics, the JM approach may facilitate individualized prognostication, though ongoing development of this approach is necessary to elucidate clinical utility.

## Methods

### Study population and enrollment

Patients with HR+/HER2- mBC receiving primary treatment at the Princess Margaret Cancer Centre were prospectively enrolled from 2018 onward. All participants provided written informed consent for serial blood collection during their standard-of-care blood work, in compliance with University Health Network (UHN) Research Ethics Board policies and the Declaration of Helsinki (REB#: 23-5123). Blood samples were collected and banked under the Liquid Biopsy Evaluation and Repository Development at Princess Margaret (LIBERATE) protocol (NCT03702309). Standard-of-care follow-up, including therapy selection, dose adjustments, radiographic evaluations, and adverse event management, was determined by the treating physician in collaboration with each patient. Discontinuation of CDK4/6i was also at the discretion of the treating physician, guided by radiographic progression, clinical progression, treatment-related adverse events, and patient preference.

### Specimen collection and handling

Serial blood samples (three Streck blood collection tubes per visit) were collected prospectively at baseline and during follow-up visits aligned with routine standard-of-care clinical appointments, timed to coincide with regular restaging radiographic assessments. Following collection, blood samples were processed through double centrifugation, separated into plasma and buffy coat, aliquoted, and stored at -80°C until analysis. For each timepoint, approximately 4 mL of plasma was sent to Guardant Health (Redwood City, California) for analysis using Guardant Reveal on the Infinity platform. The assay vendor was blinded to clinical outcomes during sequencing and analysis to ensure unbiased results.

### Guardant reveal ctDNA sequencing analysis

Up to 30 ng of cfDNA, extracted from approximately 4 mL of plasma, was partitioned based on its methylation state, then repaired, ligated with custom molecular barcodes, and amplified using polymerase chain reaction (PCR). The libraries were enriched for a significant portion (~15.2 Mb) of the DNA methylome, covering over 20,000 epigenomic regions that are differentially methylated in cancer, including 3000 regions specific to breast cancer, along with methylation control regions^31–33^. The enriched libraries were sequenced using the Illumina NovaSeq 6000 platform. Sequencing data were analyzed through Guardant Health’s proprietary bioinformatics pipeline for ctDNA detection based on predefined epigenomic signals. A methylation-based TF was estimated by normalizing cancer-specific differentially methylated regions (DMRs) against matched control regions within each sample. At each time point a TF was calculated by averaging the cancer-specific DMRs with the highest scores. The limit of detection (LoD), limit of quantification, and accuracy of the methylation-based tumor fraction were previously established in a separate set of breast, lung, colorectal, and other cancer samples^31–33^. Additionally, a description of the assays, including performance metrics, have been reported in other clinical studies^27,34^.

### Response variables and study covariates

Participants were required to have at least three temporal TF measures and no missing covariate values to be included in the analysis. Longitudinal measures between 0 and 1300 days were considered due to data sparsity issues beyond this timeframe. When reported TFs fell below the assay’s LoD, scores were replaced with 0.01%, consistent with the LoD of the methylation component of the assay. A sensitivity analysis was performed to assess the impact of using other values near the LoD, where this analysis indicated that using these replacement values did not appreciably alter the magnitude or significance levels of the relevant model parameter estimates. As mentioned, the longitudinal response variable, TF, was transformed from a percent onto the logit scale (tTF) to better adhere to model assumptions and the two time-to-event response variables were TTD and OS, respectively. All covariates included in the JM were captured at baseline and include patient age, CDK4/6i [Palbociclib vs. Ribociclib or Abemaciclib], histology [ductal vs. lobular or mixed histology], current line of therapy [1 vs. 2 or more], and prior adjuvant treatment [no vs. yes].

### Modeling approach

Technical details of the JM have been previously described^1–9,28^. The JM is divided into two sub-models, one interrogates the longitudinal data while the other interrogates the time-to-event data. Although the JM we adopted utilizes a Bayesian framework, the sub-models themselves are "frequintist" in nature. Due to the variation in temporal progression of the tTF between and within patients, a flexible cubic spline model was selected to estimate the tTF and capture its progression. To identify the best fitting model, various linear, non-linear (exponential decay, Stien-Fojo, weighted Stein-Fojo etc.), and natural, truncated, and basis cubic spline models were compared using Akaike information criteria (AIC)^35^. This investigation revealed that a basis cubic spline model with 5 knots (two boundary knots set at the minimum and maximum time points, respectively, and three internal knots set at the 25%, 50% and 75%, quantiles along the time continuum) was most optimal, and therefore, this model was used to describe the change in tTF over time. Additionally, to incorporate baseline covariates into the longitudinal modeling process, a hierarchical mixed effects model was created using the optimal spline model as a foundation. As a result, a HCSMEM was employed for the longitudinal sub-model^35,36^. For the time-to-event sub-model, a traditional CPH model, using baseline covariates as statistical controls, was utilized to analyze TTD and OS, with separate JMs constructed for each. Information from the sub-models was combined by relating the evolution of tTF to the time-to-event outcome through an association structure. Although numerous association structures are available, the most common assess the relationship between patient outcomes and: (1) the current value of the biomarker, as estimated by the longitudinal trajectory, (2) the estimated current instantaneous rate of change (slope) of the biomarker (the line tangent to the longitudinal trajectory evaluated at the most recent time point), and (3) the biomarker’s time-averaged cumulative effect—calculated by integrating the area under the trajectory followed by dividing this area by the most recent time point (i.e. the integral’s upper limit)^28,29^. Linear combinations of these association structures are also possible. We investigated the current value, slope, TACE, and their linear combinations and considered association structure p-values less than an alpha-level of 0.05 to be significant. Final model selection was guided by a combination of p-values, information criteria, and clinical interpretability. Mathematical representations of each sub-model and the association structures used to link the sub-models are supplied in the equations section of the supplementary material.

### Model development, parameter estimation, and model evaluation

The model parameters were estimated using default non-informative priors within the JMBayes2 package (Tables 2 and 3)^29^. Posterior distributions for the parameters in the OS JM were based on three chains, each with 24,000 burn-in iterations, followed by 80,000 iterations, where a thinning factor of 2 was used to alleviate autocorrelation issues. Similarly, for the TTD JM, posterior distributions were generated using three chains, each with 38,000 burn-in iterations followed by 160,000 iterations, and a thinning factor of 6 was used. Convergence of all parameter estimates was supported by the R-hat values, each falling below 1.10^37^. Since a Bayesian analysis was performed, credible intervals instead of frequentist-based confidence intervals are reported. Internal model validation to assess the reliability and predictive accuracy of the model was assessed using the BS as a metric. The BS was selected as it provided a unified measure of both discrimination and calibration, where each BS will be estimated using a model-based approach that appropriately accounts for censoring^30^. Finally, the potential bias that arises from using the same data to both test and train a model was examined using a bootstrap technique, where any bias was found to be minimal. A detailed discussion of the model validation process was provided in the results section.

## Supplementary information

Supplementary Information

## Data Availability

The datasets generated and/or analyzed during the current study are not publicly available as they contain trade secrets and intellectual property specific to Guardant Health.

## Code availability

The general R code necessary to perform the analysis is provided in the supplementary material. R Core Team (2022). R: A language and environment for statistical computing. R Foundation for Statistical Computing, Vienna, Austria. URL https://www.R-project.org/.
