# Supplementary material for: Longitudinal evaluation of serial ctDNA kinetics using joint modeling in patients with metastatic breast cancer
Source: NPJ Precis Oncol. 2026 Jun 3;10:362. doi: 10.1038/s41698-026-01498-8 (PMC13616900; doi:10.1038/s41698-026-01498-8)
Supplement: Supplementary file 1 [file 41698_2026_1498_MOESM1_ESM.pdf]

Supplementary Material

Supplementary Tables

**Model**	**DIC**	**WAIC**
TTD JM Model (Value)	2583.6	5238.8
TTD JM Model (TACE)	2653.2	2700.2
OS JM Model (Value)	2583.7	5237.8
OS JM Model (TACE)	2696.6	2681.3

Supplementary Table 1. Comparing Information Criteria of JMs Based on the Most Recent tTF Value vs the JMs Based on the Time-Averaged Cumulative Effect (Lower values indicate better model fit).

Time-to-Event JM Results for Time to Overall Survival (Time-Averaged Cumulative Effect)						
**Covariate**	**Parameter Estimate (HR)**	**SD of Estimate**	**95% CI (LB, UB)**	**HR 95% CI (LB, UB)**	**p-value**	**R-hat**
Baseline Age	0.0141 (1.01)	0.0288	(-0.044, 0.070)	(0.957, 1.07)	0.617	1.001
CDK4/6 Inhibitor: Palbociclib vs Abemaciclib or Ribociclib	-0.492 (0.612)	0.718	(-1.95, 0.867)	(0.143, 2.38)	0.498	1.006
Histology: IDC vs ILC/MDLC	-0.508 (0.602)	0.570	(-1.67, 0.570)	(0.188, 1.77)	0.371	1.003
Line of Therapy: 1st vs 2+	0.0134 (1.01)	0.501	(-1.00, 0.968)	(0.368, 2.63)	0.965	1.018
Prior Adjuvant Chemo/ET (No vs Yes)	-0.539 (0.583)	0.486	(-1.51, 0.391)	(0.222, 1.48)	0.264	1.001
Time-Averaged Cumulative Effect (tTF)	0.786 (2.19)	0.145	(0.518, 1.09)	(1.68, 2.97)	<0.0001	1.030

Supplementary Table 2. Joint Model Results for the Time-to-Event Sub-Model (Overall Survival—Time-Averaged Cumulative Effect). SD = Standard Deviation, CI = Credible Interval, LB = Lower Bound of the CI, UB = Upper Bound of the CI, IDC = invasive ductal carcinoma, ILC/MDLC = invasive lobular or mixed ductal and lobular carcinoma.

Time-to-Event JM Results for Time to Treatment Discontinuation (Time-Averaged Cumulative Effect)						
Covariate	**Parameter Estimate (HR)**	**SD of Estimate**	**95% CI (LB, UB)**	**HR 95% CI (LB, UB)**	**p-value**	**R-hat**
Baseline Age	0.013 (1.01)	0.030	(-0.05, 0.071)	(0.95, 1.07)	0.647	1.005
CDK4/6 Inhibitor: Palbociclib vs Abemaciclib or Ribociclib	-0.512 (0.60)	0.742	(-2.02, 0.889)	(0.13, 2.43)	0.499	1.004
Histology: IDC vs ILC/MDLC	-0.368 (0.69)	0.583	(-1.55, 0.744)	(0.21, 2.11)	0.534	1.006
Line of Therapy: 1st vs 2+	0.113(1.12)	0.511	(-0.91, 1.08)	(0.40, 2.95)	0.814	1.004

Prior Adjuvant Chemo/ET (No vs Yes)	-0.475 (0.62)	0.499	(-1.47, 0.482)	(0.23, 1.62)	0.339	1.001
Time-Averaged Cumulative Effect (tTF)	0.831 (2.30)	0.151	(0.540, 1.143)	(1.73, 3.14)	<0.0001	1.003

Supplementary Table 3. Joint Model Results for the Time-to-Event Sub-Model (Time to Treatment Discontinuation—Time-Averaged Cumulative Effect). SD = Standard Deviation, CI = Credible Interval, LB = Lower Bound of the CI, UB = Upper Bound of the CI, IDC = invasive ductal carcinoma, ILC/MDLC = invasive lobular or mixed ductal and lobular carcinoma.

Supplementary Equations

Longitudinal Sub-Model: Hierarchical Mixed-Effects Cubic Spline

Let $\xi_1, \ldots, \xi_M$ be a non-decreasing knot vector and let the degree of the spline be $d = 3$(cubic). The number of B-spline basis functions is then $K = M - d - 1$.

Level-1 Equation(s)

For subject i at measurement occasion j, with observation time t_{ij}, the longitudinal outcome Y_{ij} is modeled as

$$Y_{ij} = \sum_{k=1}^{K} \pi_{ki} \, B_k^{(d)}(t_{ij}) + \varepsilon_{ij}, \quad (1)$$

where $B_k^{(d)}(t)$ denotes the k-th B-spline basis function of degree d, defined via the Cox–de Boor recursion.
The B-spline basis functions are defined as follows. For degree 0,

$$B_k^{(0)}(t) = \{1, if\ \xi_k \leq t < \xi_{k+1}, 0, otherwise\},$$

and for degree $d \geq 1$,

$$B_k^{(d)}(t) = \frac{t - \xi_k}{\xi_{k+d} - \xi_k} \, B_k^{(1)}(t) + \frac{\xi_{k+d+1} - t}{\xi_{k+d+1} - \xi_{k+1}} \, B_{k+1}^{(1)}(t),$$

with the convention that terms with zero denominators are taken to be zero.

Y_{ij}: longitudinal biomarker for patient i at measurement occasion j;
t_{ij}: time of measurement j for patient i;
π_{ki}: patient-specific "growth parameters" (spline coefficients) for basis function k;
ε_{ij}: residual error, assumed $\varepsilon_{ij} \sim N(0, \sigma_\varepsilon^2)$.

Level-2 Equation(s)

Each spline coefficient π_{ki} is modeled as

$$\pi_{ki} = \beta_{k0} + \sum_{c=1}^{C} \beta_{kc} X_{ci} + e_{ki}\, , k = 1, \dots, K, \quad (2)$$

where:

- X_{ci}: *c-th covariate for patient* $i (c = 1, \dots, C)$;
- β_{k0}: *intercept for spline coefficient* k;
- β_{kc}: *effect of covariate* X_{ci} *on spline coefficient* k, *allowing a distinct set of coefficients* $\{\beta_{kc}\}$ *for each* k;
- e_{ki}: *random effect associated with spline coefficient* k *for patient* i.

Expressing the random effects in the vector

$$e_i = (e_{1i}\; e_{2i}\; \vdots\; e_{Ki}\,).$$

We assume

$$e_i \sim N_K(0, \Psi),$$

where $\boldsymbol{\Psi}$ *is a* $K \times K$ *variance–covariance matrix with generic element* $\psi_{rs} = \mathrm{Cov}(e_{ri}, e_{si})$. *In expanded form,*

$$\boldsymbol{\Psi} = \begin{pmatrix} \boldsymbol{\psi_{11}} & \boldsymbol{\psi_{12}} & \cdots & \boldsymbol{\psi_{1K}} \\ \boldsymbol{\psi_{12}} & \boldsymbol{\psi_{22}} & \cdots & \boldsymbol{\psi_{2K}} \\ \vdots & \vdots & \ddots & \vdots \\ \boldsymbol{\psi_{1K}} & \boldsymbol{\psi_{2K}} & \cdots & \boldsymbol{\psi_{KK}} \end{pmatrix}.$$

Time-to-Event Sub-Model

Let t *denote a continuous time measure for subject* i, *and define the hazard at time* t *as* $h_i(t)$. *The survival sub-model can be written as:*

$$h_i(t) = h_0(t)\, exp\{\gamma^\top w_i + \alpha\, m_i(t)\}, \quad (3)$$

where:

$h_i(t)$: *hazard of the event for subject* i *at time* t;
$h_0(t)$: *baseline hazard function evaluated at time* t;
w_i: *vector of baseline covariates for subject* i;
γ: *vector of regression coefficients for the baseline covariates* w_i;
$m_i(t)$: *latent longitudinal process from the level-1 longitudinal sub-model evaluated at time* t; *for the spline-based longitudinal model,*

$$m_i(t) = \sum_{k=1}^{K} \pi_{ki}\, B_k^{(d)}(t),$$

where $B_k^{(d)}(t)$*are the B–spline basis functions of degree* d*(with* $d = 3$*in our application) and* π_{ki}*are the subject-specific spline coefficients;*

α*: association parameter linking the longitudinal and survival sub-models; it quantifies how the current value of the longitudinal biomarker* $m_i(t)$ *influences the hazard at time* t*.*

Additional Association Structures

The Slope of the latent trajectory is defined as:

$$m_i'(t) = \frac{d}{dt} m_i(t), \quad (4)$$

and the resulting survival sub-model is:

$$h_i(t) = h_0(t)\, exp\{\gamma^\top w_i + \alpha_{slope}\, m_i'(t)\}. \quad (5)$$

The time-averaged cumulative effect (TACE) of the longitudinal trajectory is defined as:

$$\bar{m}_i(t) = \frac{1}{t}\int_0^t m_i(u)\, du, t > 0, \quad (6)$$

and the resulting survival sub-model is:

$$h_i(t) = h_0(t)\, exp\{\gamma^\top w_i + \alpha_{area}\, \bar{m}_i(t)\}. \quad (7)$$

Supplementary General R-Code

Longitudinal sub-model

```
library(nlme)     # for lme()
library(splines)   # for bs() B-spline basis functions
long_model <- lme(
  Y ~
    COV1 * bs(time, df = 6) +
    COV2 * bs(time, df = 6) +
    COV3 * bs(time, df = 6) +
    COV4 * bs(time, df = 6) +
    COV5 * bs(time, df = 6),
```

```
  random = ~ bs(time, df = 6) | ID,
  data   = long_dat,           # replace with your data frame name
  method = "ML",
  control = lmeControl(
    maxIter  = 1e6,
    msTol    = 1e-9,
    opt   = "optim",
    msMaxIter = 1e4,
    niterEM  = 1000
  )
)
summary(long_model)
```

Time-to-event sub-model

```
library(survival)   # for Surv() and coxph()
surv_model <- coxph(
  Surv(event_time, event) ~
    COV1 + COV2 + COV3 + COV4 + COV5,
  data = dat_baseline,
  x    = TRUE   # keep design matrix (needed for JMbayes2::jm)
)
```

The Joint-Model (for each association structure)

```
library(nlme)       # lme()
library(splines)    # bs()
library(survival)   # Surv(), coxph()
library(JMbayes2)   # jm()

# --- Functional form: current value ---
ff_value <- list(
  "Y" = ~ value(Y)
)

jm_value <- jm(
  Surv_object   = surv_model,
  Mixed_objects = long_model,
  time_var   = "time",
  id_var  = "ID",
  functional_forms = ff_value,
  n_iter  = 45000L,
  n_burnin  = 15000L,
  n_thin  = 3L,
  n_chains  = 3L,
  cores  = 3L
)
```

```
summary(jm_value)

# --- Functional form: slope ---
ff_slope <- list(
  "Y" = ~ slope(Y)
)

jm_slope <- jm(
  Surv_object  = surv_model,
  Mixed_objects  = long_model,
  time_var  = "time",
  id_var  = "ID",
  functional_forms = ff_slope,
  n_iter   = 45000L,
  n_burnin  = 15000L,
  n_thin  = 3L,
  n_chains  = 3L,
  cores = 3L
)
summary(jm_slope)
```

Functional form: time-averaged cumulative effect ---

```
ff_area <- list(
  "Y" = ~ area(Y)
)

jm_area <- jm(
  Surv_object      = surv_model,
  Mixed_objects    = long_model,
  time_var  = "time",
  id_var  = "ID",
  functional_forms = ff_area,
  n_iter  = 45000L,
  n_burnin = 15000L,
  n_thin  = 3L,
  n_chains  = 3L,
  cores = 3L
)

summary(jm_area)
```

Dynamic Prediction and Model Validation Code

All dynamic prediction and model validation code was adapted from:

Andrinopoulou, E.-R., Harhay, M. O., Ratcliffe, S. J., & Rizopoulos, D. (2021). *Reflections on modern methods: Dynamic prediction using joint models of longitudinal and time-to-event data.* International Journal of Epidemiology, 50(5), 1731–1743. https://doi.org/10.1093/ije/dyab047

where the authors provide an in-dept discussion of their code via the following url:

https://erandrinopoulou.github.io/EducationalCorner_JMpred/
